# Industrially relevant hydrolyzability and fermentability of sugarcane bagasse improved effectively by glycerol organosolv pretreatment

**DOI:** 10.1186/s13068-016-0472-7

**Published:** 2016-03-11

**Authors:** Fubao Fuelbiol Sun, Xiaoqin Zhao, Jiapeng Hong, Yanjun Tang, Liang Wang, Haiyan Sun, Xiang Li, Jinguang Hu

**Affiliations:** Key Laboratory of Industrial Biotechnology, School of Biotechnology, Ministry of Education, Jiangnan University, Wuxi, 214122 China; State Key Laboratory of Pulp and Paper Engineering, South China University of Technology, Guangzhou, 510640 China; Key Laboratory of Advanced Textile Materials and Manufacturing Technology, Ministry of Education, Zhejiang Sci-Tech University, Hangzhou, 310018 China; State Key Laboratory of Motor Vehicle Biofuel Technology, Henan Tianguan Group Co., Ltd, Nanyang, 473000 China; Institute of Tropical Bioscience and Biotechnology, Chinese Academy of Tropical Agricultural Sciences, Haikou, 571101 China; Forestry Products Biotechnology/Bioenergy Group, Wood Science Department, University of British Columbia, 2424 Main Mall, Vancouver, BC V6T 1Z4 Canada

**Keywords:** Atmospheric glycerol organosolv pretreatment, Sugarcane bagasse, Structural feature, Furfural and 5-hydromethyl furfural, Enzymatic hydrolysis, Ethanol fermentation

## Abstract

**Background:**

Previous work has demonstrated that glycerol organosolv pretreatment can effectively improve the hydrolyzability of various lignocellulosic substrates. This pretreatment process strategy is ideal to integrate a commercially successful lignocellulosic and vegetable oil biorefinery industry. However, industrially relevant high-solid-loading hydrolyzability and fermentability of the pretreated substrates have yet to be considered for enzyme-based lignocellulosic biorefineries.

**Results:**

In this study, an AGO pretreatment of sugarcane bagasse was evaluated with regard to the component selectivity, structural modification, hydrolyzability, and fermentation of pretreated substrates. The results showed that the AGO pretreatment presented good component selectivity, removing approximately 70 % lignin and hemicellulose, respectively, from sugarcane bagasse with a near-intact preservation (94 %) of the overall cellulose. The pretreatment deconstructed the recalcitrant architecture of natural lignocellulosic biomass, thereby modifying the structure at the macro-/micrometer level (fiber size, surface area, average size, roughness) and supermolecular level (key chemical bond dissociation) of lignocellulosic substrates towards good hydrolyzability. Notably, extraordinarily few fermentation inhibitors (<0.2 g furfural and 5-hydromethyl furfural/kg feedstock) were generated from the AGO pretreatment process, which was apparently due to the prominent role of glycerol organic solvent in protecting monosaccharides against further degradation. The 72-h enzymatic hydrolysis of pretreated substrates at 15 % solid content achieved 90 % completion with Cellic CTec2 at 10 FPU/g dried substrate. With a simple nutrition (only 10 g/L (NH_4_)_2_SO_4_) addition, the fed-batch semi-SSF of AGO-pretreated substrates (30 % solid content) almost reached 50 g/L ethanol with cellulase preparation at 10 FPU/g dried substrate. These results have revealed that the pretreated substrate is susceptible and accessible to cellulase enzymes, thereafter exhibiting remarkable hydrolyzability and fermentability.

**Conclusion:**

The AGO pretreatment is a promising candidate for the current pretreatment process towards industrially relevant enzyme-based lignocellulosic biorefineries.

**Electronic supplementary material:**

The online version of this article (doi:10.1186/s13068-016-0472-7) contains supplementary material, which is available to authorized users.

## Background

The transition from a conventional oil refinery to a “biorefinery” based on renewable lignocellulosic biomass is crucial if we are to move to a more environmentally friendly economy [1]. To make bio-based products more cost competitive with fossil-derived conventional commodities, an efficient pretreatment strategy is needed to first open up/disrupt the complex plant cell wall structure, which allows the “cellulase” enzymes to access and deconstruct the polysaccharides within the biomass into a monomeric sugar platform [2]. Although numerous potential pretreatment strategies such as dilute acid/alkaline pretreatment, steam explosion, ammonia fiber explosion (AFEX), sulfite pretreatment to overcome recalcitrance of lignocellulose (SPORL), and ionic liquid and liquid hot water pretreatment have been extensively studied, these current pretreatment techniques still pose various challenges, especially in terms of excess water use and expensive chemicals and fermentation inhibitors [3–8].

Organosolv pretreatment, in contrast, has attracted significant attention owing to the efficiency of fractionating the biomass components and the potential of scale-up in the commercially relevant biorefinery process [9–12]. However, the low-boiling-point organic solvents used in the pretreatment process have restricted the process development because of the high-pressure operation and their high volatility and flammability. In addition, the cost of these solvents is usually too high to be industrially feasible. Therefore, it is crucial to identify a high-boiling-point organic solvent with a low price for the development of an efficient organosolv pretreatment process.

Glycerol, a high-boiling-point organic solvent derived from the oleochemical industry as a by-product has become very attractive. Recently, with the increasingly flourishing biodiesel production, it is envisioned that the glycerol market will become oversupplied in the near future [13]. In this context, an atmospheric glycerol organosolv (AGO) pretreatment, using industrial glycerol derived from the oleochemical industry, was initiated in our lab [14, 15]. The AGO pretreatment removed >65 % lignin and >80 % hemicellulose while keeping cellulose (>95 %) almost intact at the level initially assessed on the wheat straw, and the AGO-pretreated wheat straw was efficiently hydrolyzed (>90 % cellulose conversion) after 24 h. Since then, numerous research groups have tested glycerol-based organosolv pretreatment on various lignocellulosic biomass. Martín et al. [16] studied the effect of glycerol pretreatment on the main components of sugarcane bagasse, showing that the glycerol acted more selectively on lignin than on xylan. Moreover, the cellulose was almost completely recovered in the pretreated solids, accounting for 72 % (g g^−1^) of the pretreated substrate. Novo et al. [17] showed that the glycerol pretreatment attained good cellulose preservation (>91 %) and lignin removal (80 %). Zhang et al. [18] reported that >96 % of the cellulose was recovered, whereas the lignin and hemicellulose removal was almost 60 and 80 %, respectively, with an acid-catalyzed glycerol organosolv pretreatment. Romaní et al. [19] found that aqueous glycerol pretreatment removed 72 % lignin from the *Eucalyptus globulus* wood and preserved an unprecedentedly high cellulose content (83 %, corresponding to 98 % of the original) in the pretreated substrate. Moreover, the pretreated substrate presented 98 % of 48-h enzymatic hydrolysis with 20 filter paper units (FPU) of Celluclast 1.5 L and 100 IU of Novozyme 188 of each gram substrate. Hundt et al. [20] realized almost complete enzymatic hydrolysis of the alkaline glycerol-pretreated wood with an enzyme loading of 15 FPU/g Cellulase (Celluclast 1.5 L) and 30 CBU/g *β*-glucosidase (Novozyme 188). Briefly, glycerol is an excellent solvent for organosolv pretreatment. Glycerol organosolv pretreatment has outstanding selectivity in a range of various natural lignocellulosic substrates, thereby effectively improving the hydrolyzability of lignocellulosic substrates [21].

However, most of the present literature has evaluated the substrate hydrolyzability at a very low (<5 %) solid content, which is far from the industrially relevant enzyme-based lignocellulosic biorefinery process [12]. Furthermore, there is currently very little information on the microbial fermentation of glycerol organosolv-pretreated substrates. Accordingly, the objective of this study is to investigate the effect of AGO pretreatment on the composition and structure of sugarcane bagasse with various modern analytical methods, i.e., scanning electron microscopy (SEM), atomic force microscopy (AFM), confocal laser scanning microscopy (CLSM), thermogravimetric analysis (TGA), and Fourier transform infrared spectroscopy (FT-IR). The hydrolyzability of pretreated substrates at a high consistency (>10 %) was then assessed with a new commercial cellulase preparation Cellic CTec2 at a relatively low enzyme loading (6 and 10 FPU/g dried substrate) to correlate its susceptibility with the commercially successful enzymatic saccharification process. Finally, a high-gravity mash ethanol fermentation was carried out with simultaneous saccharification and fermentation (SSF), SSF with prehydrolysis (semi-SSF), and fed-batch semi-SSF to describe the fermentability of AGO-pretreated substrates. Additionally, key fermentation inhibitory compounds that have been often reported were also detected, followed by a mass balance analysis.

## Results and discussion

In our initial work, the AGO pretreatment of wheat straw was performed in a 70 % glycerol liquor at 220 °C of cooking temperature and 3 h of holding time with a preliminary selection [15]. With respect to the feedstock variance, therefore, it is necessary to choose some key parameters for the sugarcane bagasse.

### Preliminary selection of pretreatment condition

#### Pretreatment temperature

**Fig. 1:**
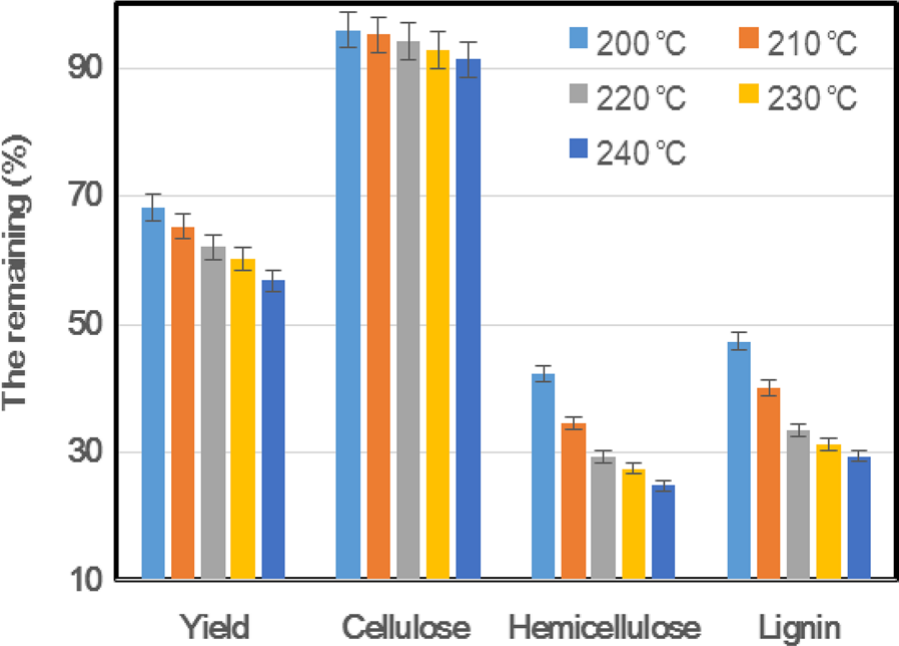
Selection of maintaining temperature for the AGO pretreatment of sugarcane bagasse. The pretreatment was performed at different temperatures for 2 h with a stirring speed of 150 rpm. The remaining means the insoluble solid fraction after the pretreatment, including its yield and main components (cellulose, hemicellulose, and lignin). Yield = [Insoluble solid (g)/Original biomass (g)] × 100; Component (cellulose, hemicellulose and lignin) (g)/the original (g) × 100

In this work, each 10 g of dried sugarcane bagasse was cooked at different temperatures for 2 h in 200 g, 70 % aqueous glycerol liquor. The pretreatment yields and main chemical components of the sugarcane bagasse are shown in Fig. 1. With the pretreatment temperature increasing from 200 to 240 °C, the pretreatment yield decreased significantly from 68 to 57 %, and the original cellulose also degraded marginally, deceasing from 96 (210) to 91 % (240 °C). When the pretreatment temperature was lower than 220 °C, lignin and hemicellulose degraded sharply from the sugarcane bagasse with the increase in pretreatment temperature. At 220 °C, the residual lignin and hemicellulose in the pretreated substrate remained at 33 and 29 %, respectively, of the original content. With a pretreatment temperature higher than 220 °C, the lignin and hemicellulose content remained almost unchanged. These results are very similar to those obtained for wheat straw in our earlier work [16]. Therefore, it is adequate to select 220 °C as the reaction temperature for the AGO pretreatment of sugarcane bagasse.

#### Pretreatment time

**Fig. 2:**
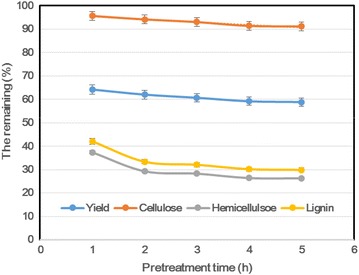
Selection of holding time for the pretreatment of sugarcane bagasse. The pretreatment was performed at 220 °C for different time with a stirring speed of 150 rpm. The definition and calculation of some parameters are as above

Figure 2 shows some effects of pretreatment time on the main components of sugarcane bagasse. With the AGO pretreatment at 220 °C from 1 to 5 h, the pretreatment yield decreased from 64 to 59 %, and the cellulose content decreased slightly from 96 (1) to 91 % (5 h). With the prolonged pretreatment time, there was a large change in hemicellulose and lignin removal. When the pretreatment time was shorter than 2 h, the lignin and hemicellulose were removed significantly from the sugarcane bagasse. At 2 h, the removal of lignin and hemicellulose reached 67 and 71 %, respectively. Thereafter, the hemicellulose and lignin remaining in the pretreated substrates were almost stable with the extended pretreatment time. Therefore, it is favorable to choose 2 h as a desirable holding time for the AGO pretreatment.

With the foregoing preliminary selection, the AGO pretreatment of sugarcane bagasse should be performed at 220 °C for 2 h in a 70 % glycerol liquor, which is very similar to that obtained for wheat straw [15]. The pretreatment removed approximately 70 % of the lignin and hemicellulose from sugarcane bagasse while maintaining the overall cellulose (94 %) almost intact. That is, the pretreated substrate had a cellulose purity of 60 %, mingled with a small amount of hemicellulose (13 %) and lignin (14 %). The result is consistent with the recent work summarized by our coworkers [21]. Such a high cellulose content in pretreated substrates is very attractive for ongoing industrially relevant high-titer bioethanol production.

#### Structural features

The foregoing AGO pretreatment technique possesses a remarkable qualification for the selection of components for natural lignocellulosic feedstock. It is interesting to feature the impact of this selection on lignocellulosic architecture. Recently, a few researchers have argued in their work on some leading pretreatments that some chemical components (lignin, hemicellulose, and acetyl group), physical properties (average size, surface area, roughness, and component redistribution), and supermolecular structures (key chemical bonds, functional groups) of the lignocellulosic biomass should be used to represent the substrate’s inherent recalcitrance to enzymatic hydrolysis [22, 23]. Accordingly, it is presumable that the AGO pretreatment should deconstruct and modify the architecture of the lignocellulosic substrate. To verify this presumption, structural features in these properties of sugarcane bagasse before and after AGO pretreatment are characterized with a series of modern analytical equipment.

### Multi-scale visualization

#### TGA

**Fig. 3:**
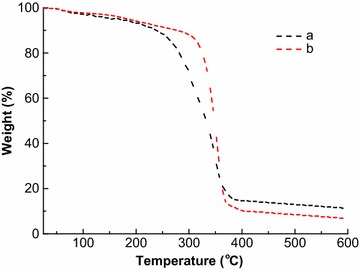
TGA curves of the sugarcane bagasse (*a*) before and (*b*) after the AGO pretreatment performed at 220 °C for 2 h with a stirring speed of 150 rpm. The weight (%) means the residual mass of the samples

A TGA of the sugarcane bagasse before and after AGO pretreatment was made, as shown in Fig. 3. The original sugarcane bagasse began to degrade approximately at 217 °C, and degraded 20 % at 282 °C and 50 % at 331 °C. Relatively, the pretreated sample started the decomposition at about 238 °C, which decomposed 20 % at 324 °C and 50 % at 348 °C. This indicates that the AGO-pretreated sugarcane bagasse had a high degradation temperature and a high thermal stability, which is attributed to partial removal of hemicellulose and lignin. Additionally, after being heated to 530 °C, both of two sugarcane bagasse samples had a different weight of some residues. The residual of AGO-pretreated substrate weighed 6.9 %, significantly less than that (11.3 %) of the original. This indicated that some lignin and other sources of ash were also removed from the sugarcane bagasse after the AGO pretreatment, resulting in a relatively low residual content [24]. In short, due to the partial removal of hemicellulose, lignin, and other components, the AGO-pretreated lignocellulosic biomass presented a high thermal stability and low ignition residue. It is confirmed that during the AGO pretreatment some main components, i.e., lignin and hemicellulose, indeed degraded/dissolved into the pretreatment liquor.

#### SEM photos

In order to understand the physical feature of AGO-pretreated substrate, some morphological changes were determined with a series of comparative SEM observations. As seen in Additional file 1: Figure S1, the original sugarcane bagasse presented a smooth and contiguous surface with compact fiber bundles. After the AGO pretreatment, a significant size reduction occurred, and the long split fasciculus was exposed outside in an orderly manner. The long fibrils were loose and fine, which had a smaller average size with a more roughness and surface area. These images were agreeable with some recent reports [25, 26]. The observation indicates that the pretreatment physically dissected the structural barrier of substrates and led to a large portion of long defibrillated fibrils with more surface area and roughness. Further, the more surface area and roughness enabled cellulase enzymes to access and attack the cellulose in defibrillated fibrils and hence an efficient enzymatic hydrolysis.

With the images enlarged, there were some irregular deposits that were noted on the uneven microfiber surface of pretreated substrates (Additional file 1: Figure S1c). However, the present observation was unclear and unsatisfactory, and further observations were necessary.

#### AFM observation

To clarify the unidentified deposits, some AFM images were further taken (see Additional file 1: Figure S2). The original sugarcane bagasse appeared flat and even with a relatively well-oriented fibrillar structure. After the pretreatment (Additional file 1: Figure S2c), there were some irregular granules and scraps inserted/dispersed on the uneven microfiber surface, forming an irregular deposit layer, which is consistent with the above SEM observation. Similar phenomena have been observed by our colleagues [27] and other researchers [25]. In our earlier work, some granules (0.1–0.2 um in diameter) and agglomerates (0.2–0.4 um in length) were observed with SEM and precipitated on the outer surface of AGO-pretreated wheat straw, which were inferred as a lignin scrap fraction and a polysaccharide fraction, respectively. They tended to form and migrate onto the outer surface of fibers during the pretreatment, owing to a lignin recondensation and/or lignin–carbohydrate complex (LCC) [27]. The formation and migration of spherical lignin deposits can expose more internal cellulose surface and probably provide more room in the substrate, allowing the entry of cellulase enzymes to the cellulose.

Three-dimensional images of the sugarcane bagasse were further taken (Additional file 1: Figure S2b, d). The root mean square (RMS) roughness (R_q_) was calculated from the topography images and recorded simultaneously with the phase images, as shown in Additional file 1: Table S1. The average roughness of sugarcane bagasse was 65.5 nm, and it increased to 104.6 nm after AGO pretreatment. It can be judged that the roughness of substrate increased indeed after the pretreatment, though the value has fallen into a wide error margin due to the substrate heterogeneity and limited sample numbers. The high roughness of lignocellulosic substrate is acknowledged to facilitate the adsorption of cellulase enzyme and then expedite the enzymatic hydrolysis [22, 23].

#### CLSM images

**Fig. 4:**
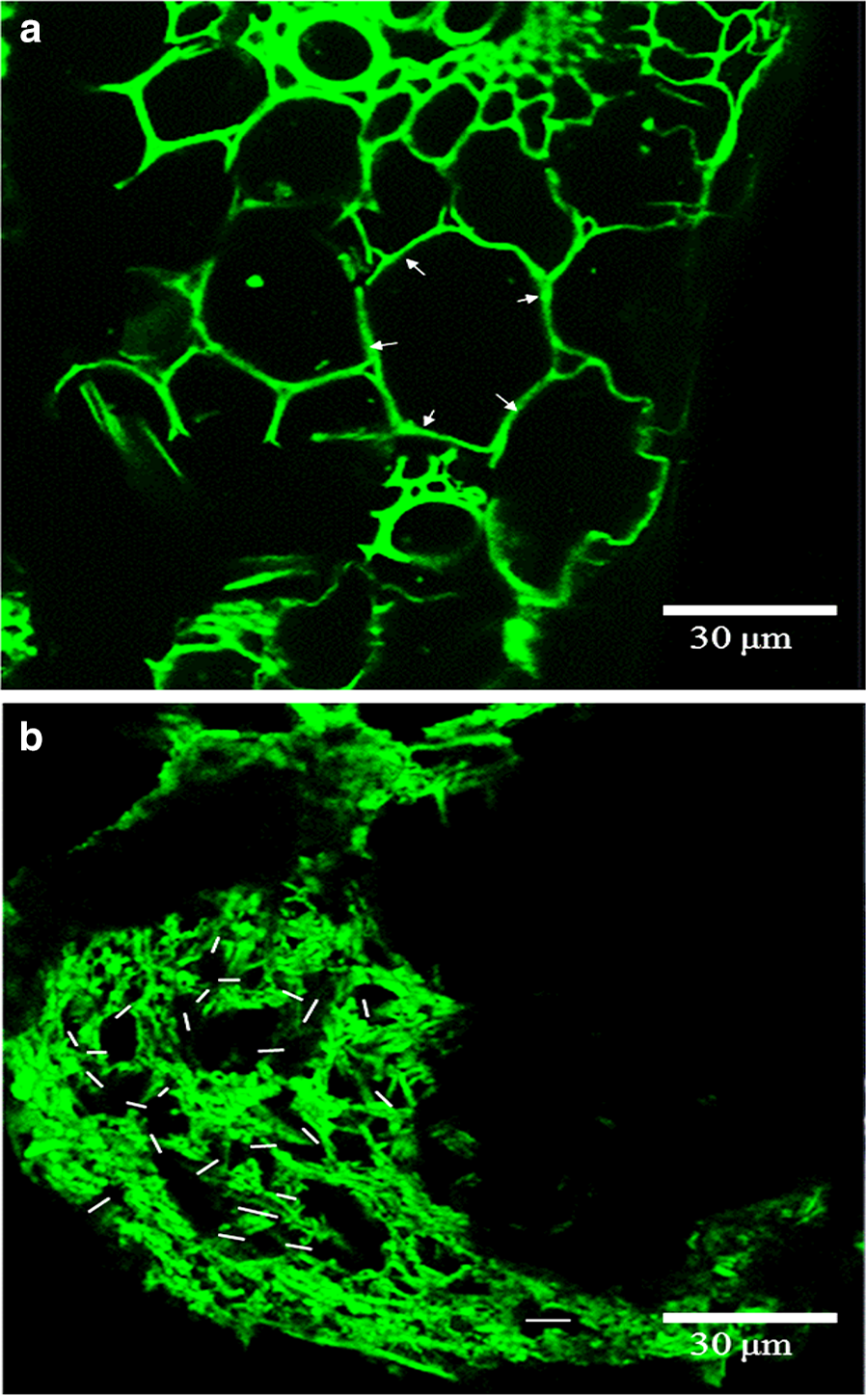
CLSM images of the sugarcane bagasse (**a**) before and (**b**) after the AGO pretreatment performed at 220 °C for 2 h with a stirring speed of 150 rpm. **a** *White arrows* point to the intact structure of lignin-rich sclerenchyma and middle lamella; **b** *white short lines* represent some chemical cuttings of the microstructure

Figure 4 illustrates CLSM images of the sugarcane bagasse (a) before and (b) after AGO pretreatment. In the original sugarcane bagasse, the lignin-rich sclerenchyma and middle lamella had an intact structure. The pretreatment resulted in a clear distortion of the whole structure at the tissue and organ levels. With a significant breakage of the microstructure of the plant cell wall, the sclerenchyma and middle lamella disintegrated, and also the primary and secondary cell walls separated from the middle lamella. These observations indicate that the AGO pretreatment has modified the recalcitrant architecture of native sugarcane bagasse, resulting in the tissue distortion, fibril swelling, and structure breakage, of which the loose fragmented structure is conducive to the enzymatic hydrolysis.

Based on the above morphological visualization, it has been demonstrated that the AGO pretreatment can indeed deconstruct the recalcitrant architecture of natural lignocellulosic biomass, with respect to the surface area, average size, component redistribution, and roughness. Moreover, it is speculated that the substrate modification is very probably due to a large dissociation of chemical bonds, especially some inter- and intramolecular hydrogen bonds responsible for the rigid and highly compact structure within biomass [23]. In order to test this, more features of sugarcane bagasse were probed at a supermolecular level.

### Mapping the supermolecular structure

Structural change of sugarcane bagasse was detected with FT-IR spectroscopy in the region of 4000–400 cm^−1^ (Additional file 1: Figure S3). Consistent with the delignification from sugarcane bagasse, the bands at 1324 cm^−1^ and the peak at 1168 cm^−1^ were all weakened. The peaks at 1604 cm, 1513, and 833 cm^−1^ all disappeared from the AGO-pretreated sample [9]. The peak at 1240 cm^−1^, assigned to β-ether bond, almost diminished, indicating maximum dissociation of the lignin polymer bond. As for hemicellulose removal, the peak at 1737 cm^−1^, representing the characteristic of the uronic ester groups of hemicellulose or hemicellulose–lignin complexes, disappeared in AGO-pretreated substrates. The absorbance peak at 1375 cm^−1^ was due to partial acetylation of hydroxyl groups in both polysaccharides and residual lignin. With a relative increase of cellulose content in the pretreated substrate, the peak at 896 cm^−1^ associated with the presence of amorphous cellulose, *β*-glycosidic linkages, and C1 group vibration was intensified after the AGO pretreatment. An intensified signal at 1200 cm^−1^ was probably related to an increase of cellulose content. The band at 1105 cm^−1^ was intensified, indicating a relative increase of the crystalline cellulose. These results indicate that the AGO pretreatment has the capacity to break down some key chemical bonds and functional groups selectively in the supermolecular structure of sugarcane bagasse, thereby resulting in the most lignin and hemicellulose removal.

For the relative crystallinity, the absorbance ratio of 1378/2900 cm^−1^, representing the total crystallinity index, increased obviously from 0.88 to 1.02 after the pretreatment. Meanwhile, the absorbance ratio of 1437/899 cm^−1^, known as the crystallinity index or lateral order index, was basically stable before (0.80) and after (0.83) the pretreatment. Also, the absorbance ratio of 1089/899 cm^−1^ means that the ratio of amorphous to crystalline cellulose had no obvious change before (0.43) and after (0.47) AGO pretreatment. It can be presumed that the AGO pretreatment led to remarkable increases of both the amorphous and crystalline cellulose contents in substrates.

### Detection of fermentation inhibitors derived from the AGO pretreatment

Numerous papers have indicated that some standing pretreatment methods—hot water [28], steam explosion [3], and organosolv pretreatment [12]—tend to produce some fermentation inhibitory compounds, such as FF and HMF. High inhibitor concentrations may severely hamper the performance of the fermenting microorganism and, in the worst case scenario, result in a non-fermentable hydrolysate. Because these inhibitory compounds have some potentially negative impacts on downstream microbial growth and biofuel production, it becomes one of the key case scenarios for a pretreatment technique to consider the formation of these by-products [28, 29]. This experiment was purposed to detect the generation of two key fermentation inhibitors, furfural (FF) and 5-hydromethyl furfural (HMF), from the AGO pretreatment. As shown in Table 1, the yield of furan derivatives was significantly different owing to the variety of natural lignocellulosic feedstock and the diversification of the pretreatment process. The total yield was surprisingly low, at <0.4 g/kg lignocellulosic feedstock for the GOP pretreatment, whereas this was generally greater than 5.0 g/kg for some leading pretreatment methods reported elsewhere. In this study, the FF and HMF content amounted to 0.30 and 0.07 g of each kilogram of sugarcane bagasse feedstock, respectively, which accounted for less than 10 % of the low production reported to date from steam explosion and ethanol organosolv pretreatment [12, 29, 30]. In this regard, the AGO pretreatment can be even competitive to some alkali pretreatments is still competitive compared with alkali pretreatments (i.e., wet oxidation and AFEX) that are generally accepted as a low furan formation (<2 g per kg lignocellulosic feedstock) [29]. A similar result was reported recently by Zhang et al. [31] on an atmospheric ethylene carbonate/ethylene glycol mixture pretreatment of sugarcane bagasse. They found that the content of xylose and furfural was very low in the pretreatment liquor, whereas no glucose and HMF were detected.

**Table 1:** Detection of some key fermentation-inhibitive compounds (g/kg substrate) produced from the AGO pretreatment and other leading pretreatments

Substrate (1 kg)	Pretreatment		Pretreatment liquor		Source
	Method	Condition	FF (g)	HMF (g)	
*Miscanthus lutarioriparious*	Liquid hot water	Pretreatment severity = 4.71	9.9	3.6	Li et al. [30]
Corn stover	Dilute acid	/	5–22	0.9–30	van der Pol et al. [31]
Corn stover	Steam explosion	210 °C, 5 min	8.6	6	Liu et al. [5]
Poplar	Steam explosion	210 °C, 4 min	4.9	0.8	Oliva et al. [3]
Poplar	60 % ethanol	180 °C, 1 h, 1.25 % H_2_SO_4_	4.4	0.9	Pan et al. [32]
Agave bagasse	50 % ethanol	160 °C, 10 min, 0.5 % H_2_SO_4_	5	3	Caspeta et al. [12]
Sugarcane bagasse	Glycerol	220 °C, 2 h	0.3	0.07	This study
Xylose	Glycerol	220 °C, 2 h	4.02	0.06	This study
Glucose			0.94	6.9	
Xylose + glucose			10.39	2.84	
Xylose + glucose	Liquid hot water		177.1	61.7	

*FF* furfural,* HMF* 5-hydromethylfurfural

To understand the formation of these furan derivatives, xylose and glucose that almost equaled the corresponding release in the liquor from sugarcane bagasse were chosen as feedstock alternatives to implement the same AGO pretreatment and liquid hot water pretreatment, respectively. For the liquid hot water pretreatment, the monosaccharide degradations were high, up to 177.1 g of FF and 61.7 g of HMF per kilogram of monosaccharide. In the glycerol system, the two inhibitors from the same amount of single or mixed monosaccharide yielded relatively low amounts. With one monosaccharide used as the lignocellulosic feedstock alternative, there was 4.0 g of FF from the xylose and 6.9 g of HMF from the glucose; 10.4 g of FF and 2.8 g of HMF were formed when these two monosaccharides were mixed together. Even though the yield from the monosaccharide was low, it was still tens times higher than that from the natural lignocellulosic feedstock (Table 1). The data indicate that the free monosaccharide was stable and did not tend to degrade into furan derivatives when suffering from the high temperature in the glycerol organosolv phase compared with the water phase. Apparently, it was not as easy to form sugar degradation products from the lignocellulosic biomass as from the monosaccharide. Alternatively, the glycerol organic solvent was probably involved in the degradation of hemicellulose/cellulose into the main oligosaccharides or xylosides/glucosides, other than the common monosaccharides formed during water-based (dilute acid, liquid hot water, and steam explosion) pretreatment [31]. The pure glycerol organic solvent can play a very prominent role in protecting monosaccharides against further degradation by the formation of xyloside/glucoside during the AGO pretreatment, thus inevitably contributing to a low furan derivative. This helps explain why extensively reported organosolv pretreatment has produced large fermentative inhibitors in the pretreatment liquor as the pretreatment solvent is mixed with high water content (20–40 %, v/v) [12]. It can be deduced that sugar polymer and organic solvent phase are mainly responsible for the extraordinarily low formation of fermentation inhibitors from natural lignocellulosic biomass. In short, the AGO pretreatment can result in extremely low fermentation inhibitory products from natural lignocellulosic biomass, which are very helpful for the downstream enzymatic hydrolysis and microbial fermentation.

### Hydrolyzability of AGO-pretreated substrates

**Fig. 5:**
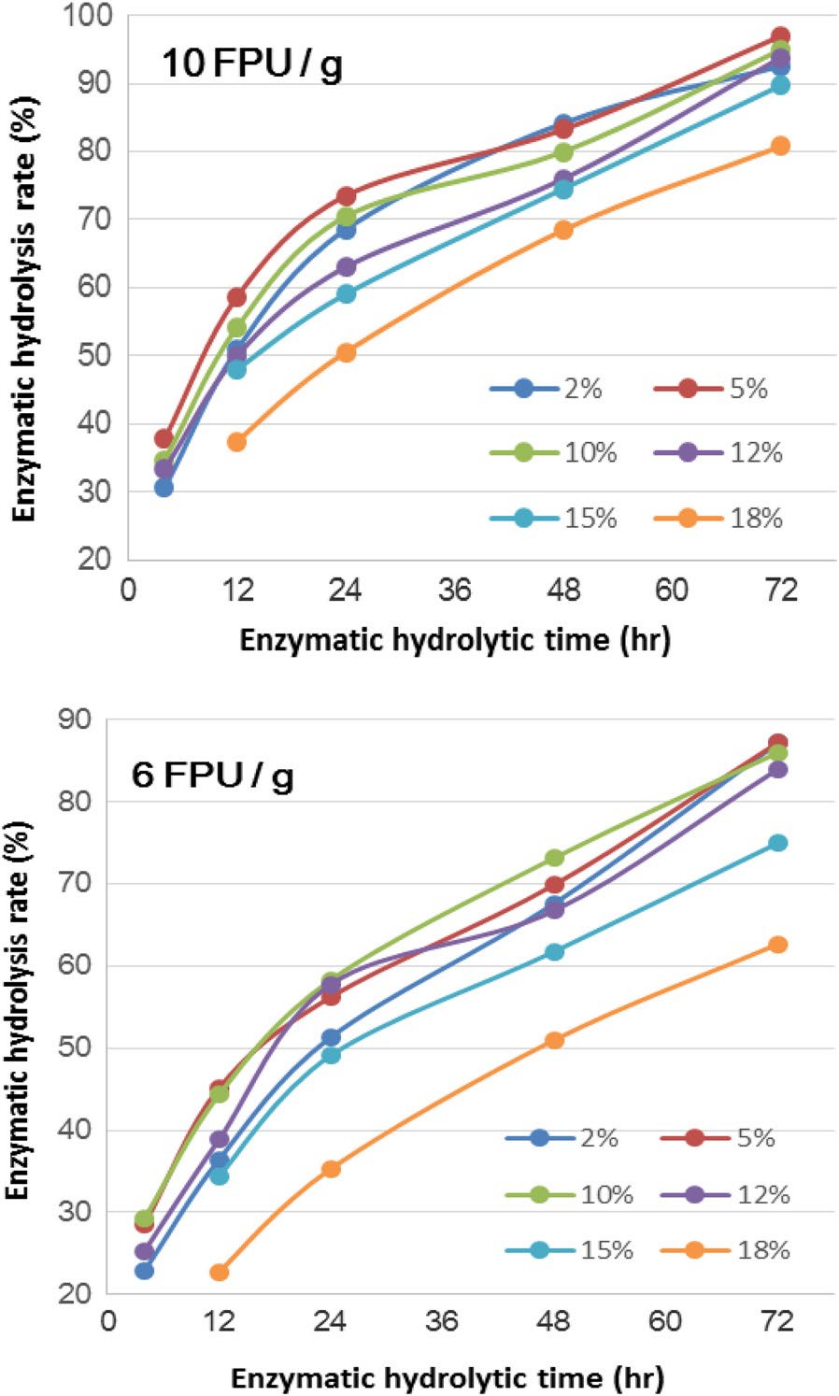
Enzymatic hydrolysis of glycerol organosolv-pretreated (220 °C, 2 h) sugarcane bagasse with Cellic CTec2 at 6 FPU/g dried substrate and 10 FPU/g dried substrate, respectively, at different solid contents [2, 5, 10, 12, 15, and 18 % (w/v)]

The above result has demonstrated that the AGO-pretreated substrate is extremely susceptible and accessible to cellulase enzymes, inclusive of no significant inhibition. In this experiment, therefore, an impact of the AGO pretreatment on downstream enzymatic hydrolysis was initially investigated with regard to the benefit of a pretreatment method for biofuel production [4, 6]. Given that economical sugar production at a high concentration is typical of the enzyme-based biorefinery process, the enzymatic hydrolyzability of sugarcane bagasse undergoing the AGO pretreatment was assessed at a high substrate consistency with commercial cellulase preparation, Cellic CTec2, at a relatively low enzyme loading, as shown in Fig. 5. At 10 FPU/g dried substrate, all substrates presented a fast enzymatic hydrolysis, and the 72-h enzymatic hydrolysis of pretreated substrate at less than 15 % solid concentration exceeded 90 %. At 6 FPU/g dried substrate, the 72-h enzymatic hydrolysis of substrates (≤12 % consistency) was greater than 80 %. Compared with our recent report on AGO-pretreated wheat straw, the hydrolyzability of AGO-pretreated sugarcane bagasse was significantly good, which was probably due to the difference in cellulase preparation and lignocellulosic variety [21]. The hydrolyzability of AGO-pretreated substrate is outstanding compared with that from steam explosion [5] and AFEX [7]. These data demonstrate that the AGO pretreatment has enabled good hydrolyzability in sugarcane bagasse.

Interestingly, it is apparent that the new cellulase preparation Cellic CTec2 has a remarkable qualification in its toleration to high solid consistency. There is no significant adverse effect on the enzymatic hydrolysis of substrate until reaching 12 % solid content, suggesting that the conventional substrate concentration (2 %) is inapplicable to evaluate the hydrolyzability of a substrate or the efficiency of a new commercial cellulase preparation (e.g., Accellerase^®^ DUET, Accellerase^®^ TRIO™ and Cellic CTec3) [6]. Moreover, the solid content of 5–10 % is probably reasonable for the future industrially relevant evaluation.

### Fermentability of AGO-pretreated substrates

**Fig. 6:**
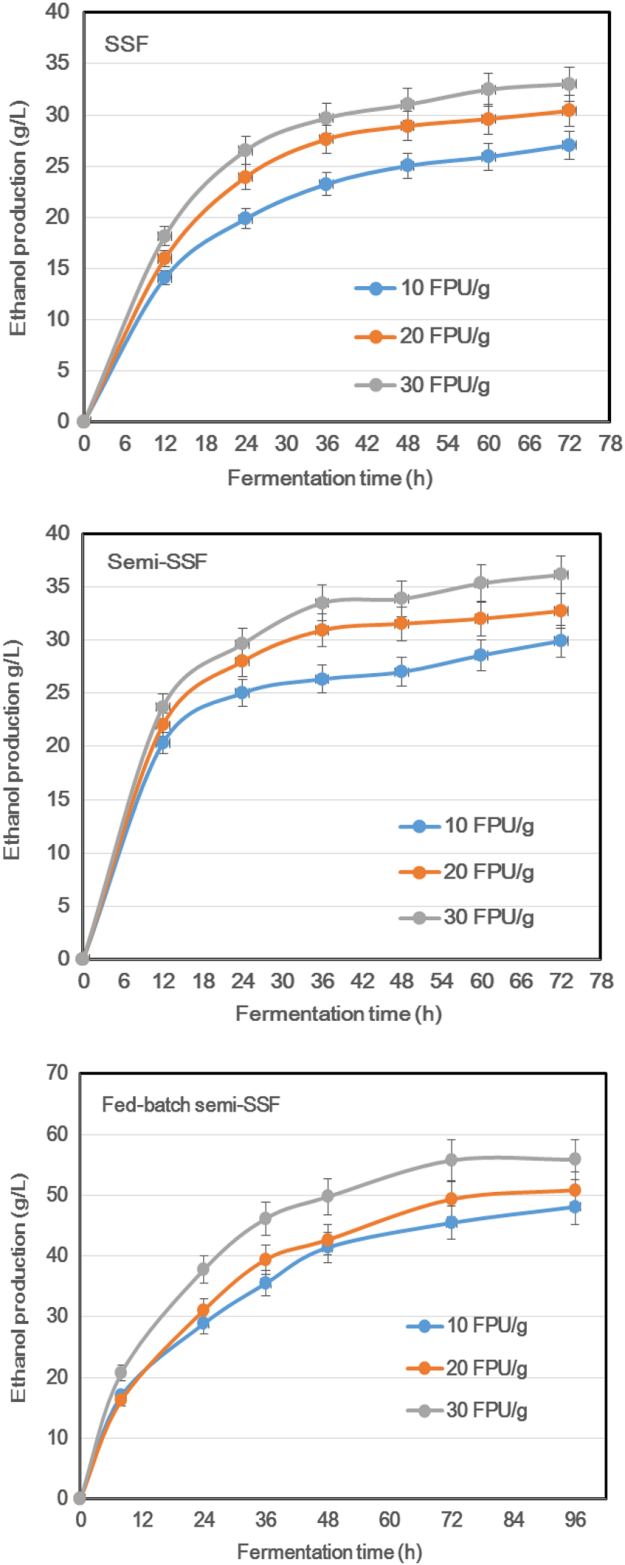
Ethanol fermentation of glycerol organosolv-pretreated (220 °C, 2 h) sugarcane bagasse at an initial solid content of 15 % (w/v). The enzyme loading of cellulase Cellic CTec2 was 10, 20, and 30 FPU/g dried substrate. For the SSF, the fermentation (initial pH 4.8, inoculation size 10 %, and shaking speed 120 r/min) was carried out at 37 °C for 72 h. For the semi-SSF and fed-batch semi-SSF, the prehydrolysis was made at 50 °C for 24 h. For the fed-batch semi-SSF, the other part of dried pretreated substrate (as much as total dried substrate used for the prehydrolysis) was divided into three aliquots and fed simultaneously with some corresponding cellulase enzymes into the fermenter after 8, 24, and 36 h of fermentation to ensure an equivalent 30 % (w/v) solid content

The foregoing result on enzymatic hydrolysis has indicated that there is a significant inhibition of the enzymatic hydrolysis occurring at >15 % solid content, owing to the slurry’s rheological property and sugar products’ feedback effect [5]. Nevertheless, the high solid loadings (≥15 %) in the unit operations of lignocellulose conversion contribute to an increased sugar and ethanol concentration and thus a low production and capital cost [12]. Accordingly, the experiment was implemented to evaluate the fermentability of pretreated substrates at an initial 15 % solid content by adopting SSF, Semi-SSF, and fed-batch semi-SSF. As shown in Fig. 6, with the SSF for 72 h, the ethanol production from AGO-pretreated substrates was 27.0 g/L at 10 FPU/g, 30.4 g/L at 20 FPU/g, and 33.0 g/L at 30 FPU/g of cellulase enzyme loading, respectively. At 24-h fermentation, there was less than 1 g/L of the glucose remaining in the fermentation broth (data not shown), indicating that low efficiency of the enzymatic hydrolysis probably hampered the ethanol fermentation. Accordingly, a 24-h prehydrolysis before the SSF—namely the semi-SSF—was adopted to strengthen the enzymatic hydrolysis. The 72-h ethanol production reached 29.9 g/L at 10 FPU/g, 32.7 g/L at 20 FPU/g, and 36.1 g/L at 30 FPU/g. Compared with the SSF, the ethanol production with the semi-SSF was enhanced by approximately 10 %. The result is unsatisfactory; a titer of 4 % in the cellulosic ethanol fermentation broth is the minimum requirement for an economical distillation process. Based on the semi-SSF, one-third of the total dried pretreated substrate, together with the corresponding enzyme loading, was fed into the fermenter after 8, 24, and 36 h of fermentation, respectively, thus attaining a final equivalent content of 30 % solid content. The results showed that the 72-h ethanol production achieved 45.4 g/L at 10 FPU/g, 49.3 g/L at 20 FPU/g, and 55.7 g/L at 30 FPU/g, each increasing 60–70 % compared with their respective initial SSF. The ethanol productivity (based on both enzymatic hydrolysis and fermentation) correspondingly reached 0.47, 0.51, and 0.58 g/L/h. To date, there are very few studies on >50 g/L of the bioethanol production and >0.5 g/L/h of the productivity, although there are some good examples of ethanol yield (200 ± 20 g/kg feedstock) [7, 8, 28]. Consequently, the fermentability of AGO-pretreated lignocellulosic substrate is good and industrially relevant.

Additionally, a simple nutrient (only 10 g/L NH_4_SO_4_) was added into the fermentation slurry. The good fermentation performance of AGO-pretreated substrates is likely because the AGO pretreatment requires moderate washing steps, thereby preserving essential biomass nutrients for fermentation. The other reason is that the AGO pretreatment, like AFEX pretreatment, does not produce a highly inhibitory pretreatment mixture [7]. In short, the AGO pretreatment is promising as an efficient technology to convert plant-derived polysaccharides to bioethanol.

### Mass balance analysis

**Fig. 7:**
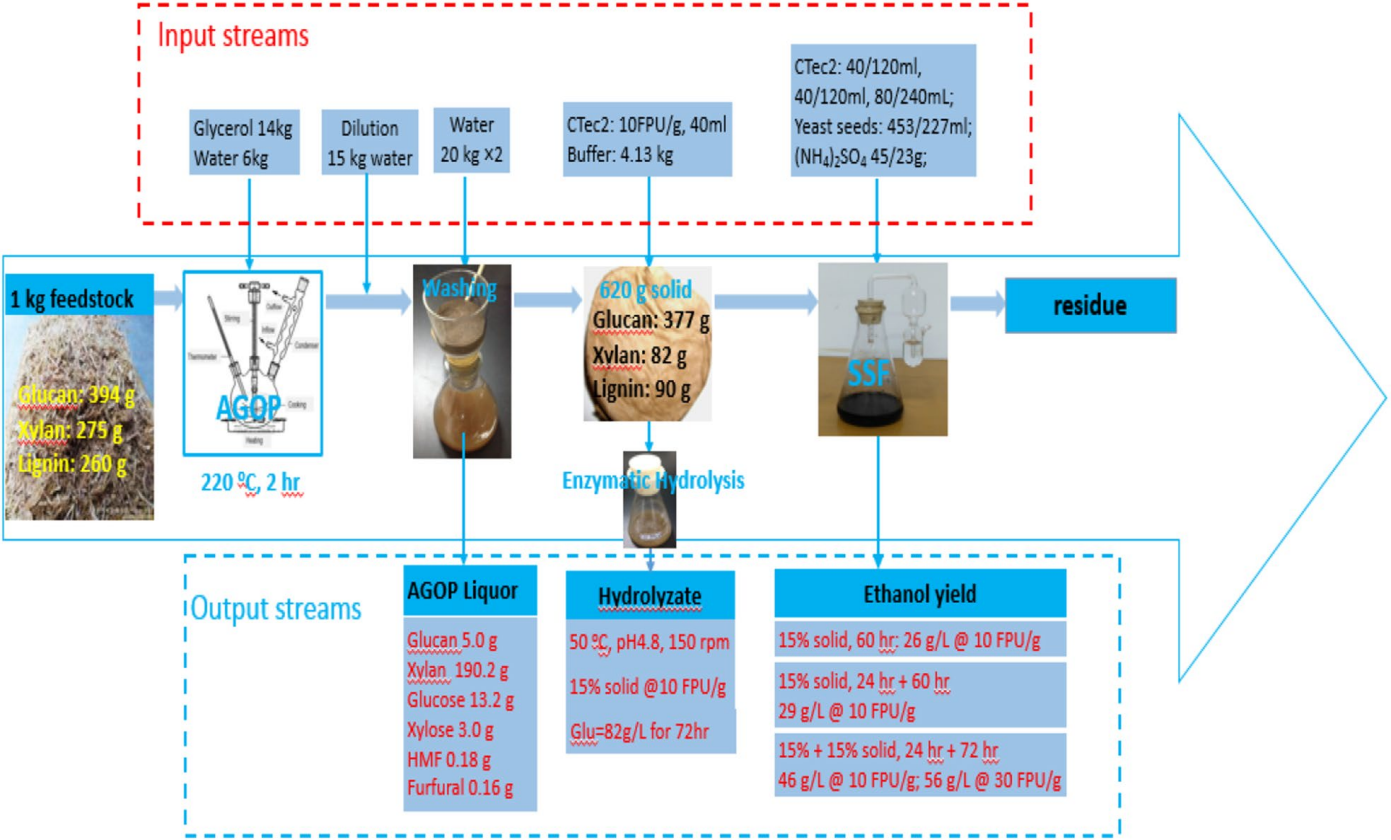
Mass balances of AGO pretreatment, enzymatic hydrolysis, and ethanol fermentation. The pretreatment was performed at 220 °C for 2 h with initial solid-to-liquid ratio of 10 g/200 g. The enzymatic hydrolysis was made at 15 % (w/v) solid content for 72 h with Cellic CTec2 at 10 FPU/g dried substrate. The high-consistency ethanol fermentation was implemented at 37 °C, 120 rpm for 72 h with Cellic CTec2 using SSF (15 %, w/v), semi-SSF (with an additional 24-h prehydrolysis at 50 °C, 120 rpm), and fed-batch semi-SSF (another dried substrate, equivalent to the substrate weight of 15 % initial solid content, was trisected and fed at 8, 24, and 36 h)

The analysis presents an entire workflow based on the laboratory test of AGO pretreatment, enzymatic hydrolysis (@ 10 FPU/g with 15 % solid content), and fed-batch semi-SSF. As shown in Fig. 7, one kilogram of sugarcane bagasse was mixed with 14 kg of industrial glycerol and 6 kg of tap water in the cooker for the pretreatment. The water was evaporated and cooled at the temperature-elevating stage for recycling use. Actually, the glycerol in the AGO pretreatment liquor can be recycled almost ten times with simple precipitation and centrifugation. In the pretreatment liquor, most of the glucan surviving from the pretreatment existed in the form of glucose, apparently accounting for 2/3 of the total cellulose loss. Strikingly, the xylan that split away from the undissolved solid existed in a very slight form of xylose (<2 %), predominantly the xylose polymers. This slight monosaccharide is responsible at least partly for the low inhibitory pretreatment mixture of FF and HMF. The AGO pretreatment was able to remove most of the lignin from sugarcane bagasse, generating a solid fraction enriched with glucan (60.8 %). The undissolved solid has outstanding enzymatic hydrolyzability, achieving 81.7 % of the 72-h glucan hydrolysis (@ 9 % glucan loading) with Cellic CTec2 at 15 FPU/g glucan. The final glucose content attained 82 g/L in the hydrolysate. It can be envisioned that the enzyme loading to complete the same enzymatic hydrolysis would be reduced to 5 FPU/g glucan with an optimal use of some new cellulase enzyme preparations/cocktails (i.e., Cellic^®^ CTec3, Cellic HTec3 and AA9) [33]. The good hydrolyzability of substrates enabled by the AGO pretreatment can contribute to an extremely significant step toward the more cost-effective commercial-scale production of cellulosic ethanol. Based on the high-solid-loading enzymatic hydrolysis, the fermentability of AGO-pretreated substrates was verified with an initial 15 % solid content at 10 and 30 FPU/g dried substrate. The cellulosic ethanol yield was achieved approximately 50 g/L using a fed-batch semi-SSF of the total 96 h. In this scenario, the xylose derived from the hydrolysis of hemicellulose residual (13.2 %) in the pretreated substrate accumulated to almost 40 g/L (data not shown) because the thermally tolerant alcohol yeast is not capable of consuming it. Therefore, it is necessary to consider the xylose-to-ethanol conversion in the future work for the economic production of cellulosic ethanol at a commercial scale [8]. Despite an interestingly high ethanol production (~50 g/L), the cellulose-to-ethanol conversion by fed-batch semi-SSF was still very low—43.5 % at 10 FPU/g and 53.4 % at 30 FPU/g. Unlike other standing pretreatment methods [4], the glycerol that is entrapped in the undissolved solid (<15 % substrate weight) is likely to have a very low impact on the downstream enzymatic hydrolysis and microbial fermentation (30 % solid content) because it is a natural carbon source of many microorganisms [24]. Accordingly, it can be assumed that the low ethanol conversion originates exclusively from the substandard enzymatic hydrolysis. In the future work, it is necessary to carry out considerable research to streamline the enzymatic hydrolysis process to make the bioethanol fermentation cost effective.

These results on the hydrolyzability and fermentability correlate well with the aforementioned physicochemical properties (compositional change and structural feature) of AGO-pretreated substrates. Based on the above analysis, it is evident that the glycerol organosolv pretreatment is promising for the current pretreatment. Additionally, this pretreatment process strategy is ideal for integration into a commercially successful lignocellulosic and vegetable oil biorefinery industry.

## Conclusions

The AGO pretreatment has presented good selectivity on sugarcane bagasse. The pretreatment can contribute to an amazing physiochemical modification of lignocellulosic biomass towards superior susceptibility and accessibility to cellulase enzymes, with a high cellulose purity (~60 %). Notably, there are extremely low amounts of fermentation-inhibitive compounds (i.e., <0.40 g FF and <0.10 g HMF kg^−1^ feedstock) generated in the pretreatment process. The undissolved solid has outstanding enzymatic hydrolyzability, achieving 90 % of the 72-h enzymatic hydrolysis at 15 % solid content with Cellic CTec2 at 10 FPU/g dried substrate. With a simple nutrition (only 10 g/L (NH_4_)_2_SO_4_) addition, the fed-batch semi-SSF of AGO-pretreated substrates reached almost 50 g/L ethanol (30 % solid content) with the cellulase preparation at 10 FPU/g dried substrate. The AGO pretreatment is promising for the current pretreatment towards industrially relevant enzyme-based lignocellulosic biorefineries. Further improvements in overall ethanol yield and titer are required to increase the commercial attractiveness of this technology.

## Methods

### Materials

Sugarcane bagasse was obtained from Guangxi Province, China. It was dried to constant weight at 60 °C and then stored in a polyethylene plastic container. The main components of sugarcane bagasse were as follows: 39.4 % glucan, 27.5 % xylan, and 26.0 % lignin. Industrial glycerol was of commercial grade (95 % purity), purchased from a chemical plant in Jiangsu Province, China. It was diluted to 70 % for use in the experiment. Cellulase Cellic CTec2 (150 FPU/g) was a generous gift from Novozymes (China) Investment Co. Ltd.

### AGO pretreatment process

In a typical run, 10 g of dry sugarcane bagasse was directly mixed with 200 g of aqueous glycerol (70 % industrial glycerol) in a three-necked round-bottom flask as reported in our earlier work [14, 15]. A condenser was fitted in the right-hand neck of the flask for reflux, a thermocouple wire was passed through the left-hand neck to measure the temperature, and a stirrer was fitted into the middle neck of the flask to ensure full mixture stirring. The flask was heated at different combinations of temperature and time ranging from 200 to 240 °C for 1–5 h. After heating to the desired temperature for the specified period of time, the mixture was allowed to cool to approximately 120 °C. One hundred fifty grams of distilled water was then added. The mixture was stirred vigorously to ensure thorough disintegration. The insoluble solid fraction was then separated by filtration through a G_3_ glass filter (100 mL, pore size 15–40 μm) and washed twice with 200 g of distilled water, and the insoluble solid fraction was divided into two parts. One part was conserved in a sealed bag at 5 °C for further enzymatic hydrolysis, and the other was dried at 60 °C to determine the pretreatment yield (the percentage of total solids recovered from the original feedstock), main components (the percentage of cellulose/hemicellulose/lignin in the insoluble solid fraction to that of the original feedstock), and structural change. The pretreatment liquor was collected to detect the main composition, including glucose, xylose, FF, and HMF. In the experiment on AGO pretreatment, 0.40 g of glucose and 2.0 g of xylose were used alone and together, respectively, as an alternative to the 10 g of dry sugarcane bagasse under the same condition mentioned above. The liquid hot water treatment of 0.40 g of glucose and 2.0 g of xylose alone and together, respectively, was performed in a batch reactor (six-parallel acid digestion bomb of 200 mL, SS 316 with Teflon liner) under the conditions mentioned above.

### Enzymatic hydrolysis of the resulting fiber fractions

The wet solid substrate from the AGO pretreatment was put at different solid loadings into a 150-mL flask and suspended quickly with 50 mL citrate buffer (0.05 M, pH 4.8) with an Cellic CTec2 loading of 10, 20, or 30 FPU/g dried substrate [14, 15]. The experiment was performed at 150 rpm in a rotary shaker at 50 °C for 72 h, wherein samples were withdrawn at interval time and centrifuged at 10,000 rpm for 10 min to detect the total reducing sugar content. Enzymatic hydrolysis (%) = Total reducing sugars in the hydrolysate (g) × 0.9/[Cellulose (g) + Hemicellulose (g)] in substrates. Experiments with each sample were performed in duplicate, with the average value reported. The average relative standard deviation was below 3 %.

### Microorganism and seed culture preparation

Angel^®^ thermal tolerance alcohol active dry yeast (Angel Yeast Co., Ltd., Yichang, China) was used in this study [5, 28]. Seed cultures were prepared in a 150-ml flask with 50 ml of medium containing (g/L) yeast extract 5, tryptone 5, glucose 50, KH_2_PO_4_ 1, MgSO_4_ 0.3, and NH_4_Cl 2, at 37 °C and 120 rpm for 12 h. The seed liquid was then inoculated into the fermentation slurry at 10 % (v/v) of the inoculation size.

### Ethanol fermentation

In the SSF, semi-SSF, and fed-batch semi-SSF, wet substrate from the undissolved solid fraction was used for the initial 15 % solid content. The wet solid (7.50 g dry weight) was placed into a 250-mL flask and suspended quickly with some citrate buffer (0.05 M, pH 4.8) [including 0.5 g (NH_4_)_2_SO_4_] to acquire 15 % solid content (w/v). After the sterilization, the cellulase was added into the slurry at 10, 20, or 30 FPU/g substrate. For the SSF, yeast seed liquid was added simultaneously with the cellulase preparation before the start of fermentation. For the semi-SSF, some cellulase preparations were first added into the slurry and hydrolyzed at 50 °C and 120 rpm for 24 h. The seed liquid was then inoculated in the broth after rapid cooling to 37 °C. For the fed-batch semi-SSF, the dried substrate (constant weight at 105 °C), amounting to one-third of the initial total dried pretreated substrate, was fed at 8, 24, and 36 h of the semi-SSF time, together with the corresponding enzyme loading. All fermentation experiments were conducted at 37 °C for 72 h with a shaking speed of 120 rpm. Samples were taken at different time points and centrifuged at 10,000 rpm for 10 min. Supernatants were filtered through a 0.22-μm Whatman syringe filter. Compositions (glucose, xylose, and ethanol) of fermentation broth were determined by HPLC [5, 28]. All of these experiments were conducted with two replicates.

### Structural characterization

A series of analytical techniques were employed as reported recently by us [21]. Thermal stability of the sample was analyzed using thermogravimetric analysis (TGA-STDA851e, Mettler-Toledo, Switzerland). Morphological Changes were observed with a scanning electron microscope (SEM), Quznfa-200 (FEI, Netherlands), an atomic force microscope (AFM), and a confocal laser scanning microscope (CLSM), LSM 710 (Zeiss, Germany). FT-IR spectra of the samples were recorded on an FT-IR spectrophotometer (NICOLET NEXUS 470, Thermo Nicolet, US) from finely ground samples (1 %) in KBr pellets in the range of 4000–400 cm^−1^.

### Analytical procedures

The total reducing sugar content was determined by the standard 3,5-dinitrosalicylic acid method [33]. The main component (cellulose, hemicellulose, and lignin content) was determined by the two-step acid hydrolysis method (NREL) [34]. The wavelength of 240 nm was used for the determination of acid-soluble lignin. A muffle furnace was used at approximately 600 °C to indirectly measure the acid-insoluble lignin. The Chromaster HITACHI HPLC system equipped with an Aminex HPX-87H column (300 × 7.8 mm, Bio-Rad, US) was used at the column temperature of 65 °C, with 5 mM H_2_SO_4_ as the mobile phase at a flow rate of 0.6 mL min^−1^ to detect the monosaccharides, ethanol, and sugar degradation products (FF and HMF). A refractive index detector (RID) and a diode array detector (DAD) were used in series. The RID was used to measure the monosaccharides (glucose, xylose, and arabinose) and ethanol. The DAD was used to detect the key sugar degradation products (FF and HMF) with a detection wavelength of 280 nm. The entire analytical determination was performed in duplicate. The average relative standard deviation was below 6 %.

## Additional file

10.1186/s13068-016-0472-7 The substrate roughness measured from AFM images. **Figure S1.** SEM images of the sugarcane bagasse before and after the AGO pretreatment. **Figure S2.** AFM images of the sugarcane bagasse before and after the AGO pretreatment. **Figure S3.** FT-IR spectra of the sugarcane bagasse before and after the AGO pretreatment.

## Abbreviations

AGO: atmospheric glycerol organosolv
SF: simultaneous saccharification and fermentation
semi-SSF: SSF with prehydrolysis
AFEX: ammonia fiber explosion
SPORL: sulfite pretreatment to overcome recalcitrance of lignocellulose
FF: furfural
HMF: 5-hydromethyl furfural
SEM: scanning electron microscopy
AFM: atomic force microscopy
CLSM: confocal laser scanning microscopy
TGA: thermogravimetric analysis
FT-IR: Fourier transform infrared spectroscopy
FPU: filter paper unit
LCC: lignin–carbohydrate complex
CrI: crystallinity index
RMS: root mean square
