# Supplementary material for: Industrially relevant hydrolyzability and fermentability of sugarcane bagasse improved effectively by glycerol organosolv pretreatment
Source: Biotechnol Biofuels. 2016 Mar 11;9:59. doi: 10.1186/s13068-016-0472-7 (PMC4788952; doi:10.1186/s13068-016-0472-7)

Supplementary Materials

Industrially relevant hydrolyzability and fermentability of sugarcane bagasse improved effectively by glycerol organosolv pretreatment

Fubao Fuelbiol Sun[1*], Xiaoqin Zhao[1,2], Jiapeng Hong[1,3], Yanjun Tang[3], Liang Wang[1,4], Haiyan Sun[5], Xiang Li[1], Jinguang Hu[6]

[1] Key Laboratory of Industrial Biotechnology, Ministry of Education, School of Biotechnology, Jiangnan University, Wuxi 214122, China

[2] State Key Laboratory of Pulp and Paper Engineering, South China University of Technology , Guangzhou 510640

[3] Key Laboratory of Advanced Textile Materials and Manufacturing Technology, Ministry of Education, Zhejiang Sci-Tech University, Hangzhou 310018, China

[4] State Key Laboratory of Motor Vehicle Biofuel Technology, Henan Tianguan Group Co., Ltd, Nanyang 473000, China

[5] Institute of Tropical Bioscience and Biotechnology, Chinese Academy of Tropical Agricultural Sciences, Haikou 571101, China

[6] Forestry Products Biotechnology/Bioenergy Group, Wood Science Department, University of British Columbia, 2424 Main Mall, Vancouver BC, V6T 1Z4, Canada;

*Corresponding Author

Fubao Fuelbiol Sun (FFS), PhD, Assoc Prof; Research Center of Bioresource and Bioenergy, School of Biotechnology, Jiangnan University; 1800 Lihu Road, Wuxi 214122, China; Tel: 86-510-85327026; Fax: 86-510-85327026; fubaosun@jiangnan.edu.cn; sunfubao@hotmail.com

Co-authors:

Xiaoqin Zhao (XZ), xiaoqinfuelbiol@163.com

Jiapeng Hong (JH), hongjp0303@163.com

Yanjun Tang (YT), tangyj@zstu.edu.cn

Liang Wang (LW), wangliangfuelbiol@126.com

Haiyan Sun (HS), sunhaiyan@itbb.org.cn

Xiang Li (XL), lixiangfuelbiol@126.com

Jinguang Hu (JHu), jinguang@interchange.ubc.ca

Table S1 Root mean square values of roughness measured from AFM images given in nanometer and the average value calculated from the three images

Sample	Roughness (nm)			Average (nm)
	1	2	3	
Original	41.8	67.5	87.1	65.5
Pretreated	86.4	137	90.3	104.6

Figure S1 SEM images of the sugarcane bagasse (a) before, (b) after and (c) the AGO pretreatment performed at 220 °C for 2 h with a stirring speed of 150 rpm. Green broken circles denote the irregular deposit.

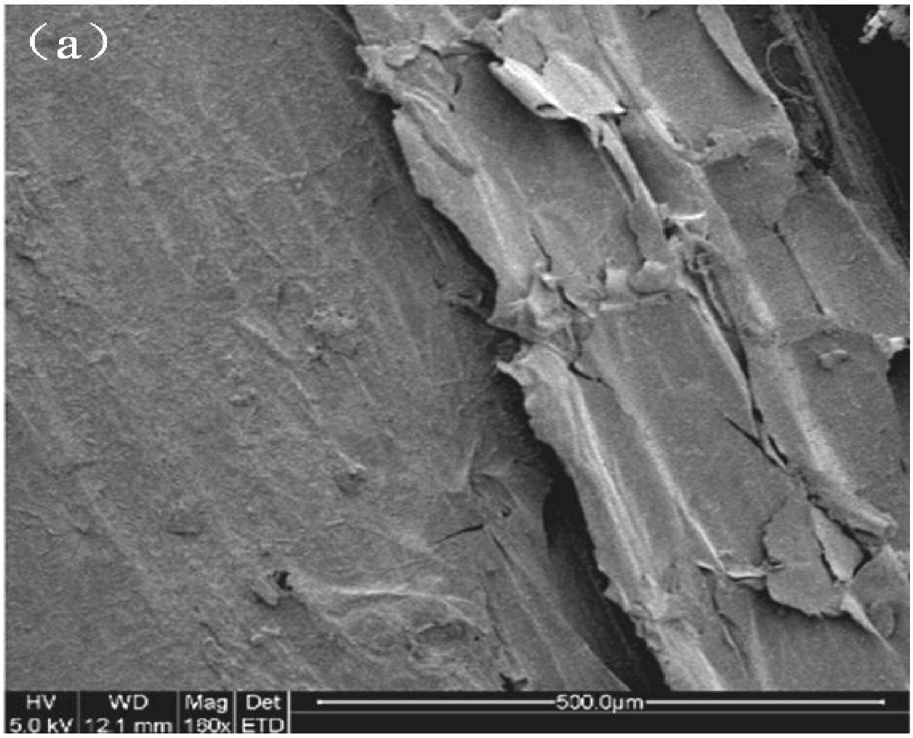

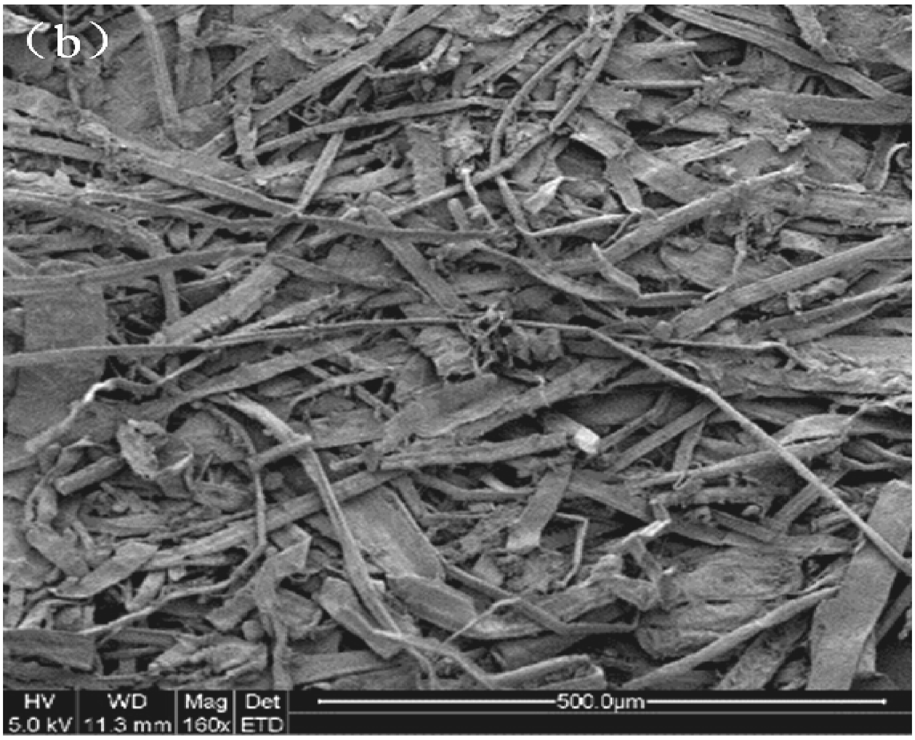

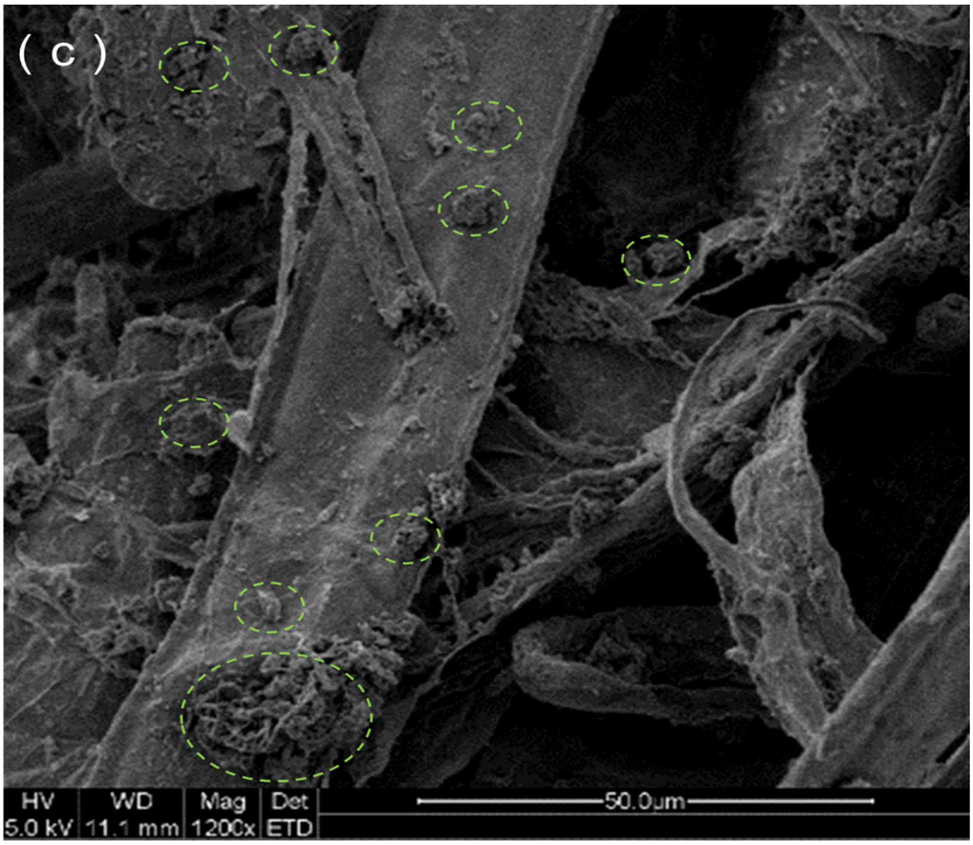
(c)
HV
5.0 kV
WD
11.1 mm
Mag
1200x
Det
ETD
50.0μm

Figure S2 AFM-phase contrast images of the sugarcane bagasse before (a, b) and after (c, d) the AGO pretreatment performed at 220 °C for 2 h in with a stirring speed of 150 rpm. The images (a) and (c) depict the wall surface, and (b) and (d) are the corresponding 3D images of (a) and (c). White and green broken circles denote the spherical lignin deposits and lignin-carbohydrate complex (LCC), respectively.

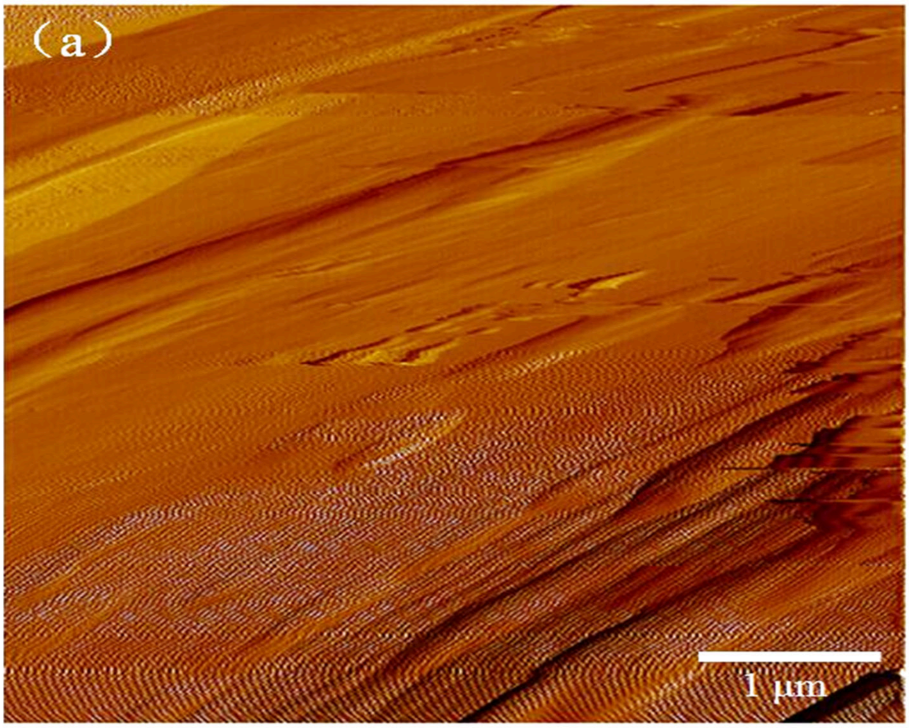

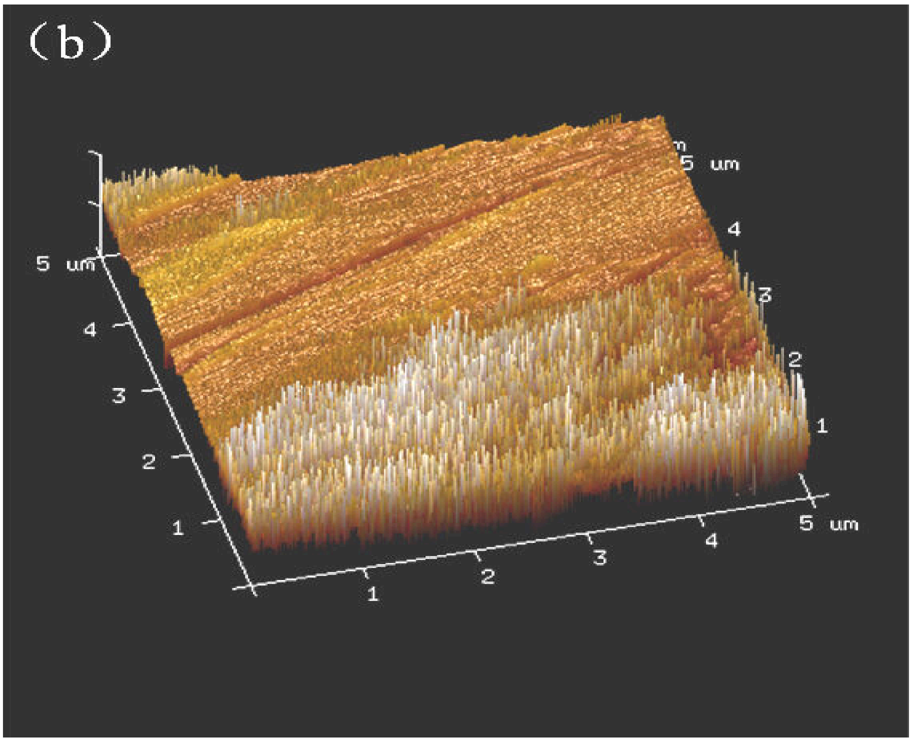
（b）
5 um
4
3
2
1
1
2
3
4
5 um
5 um
4
3
2
1

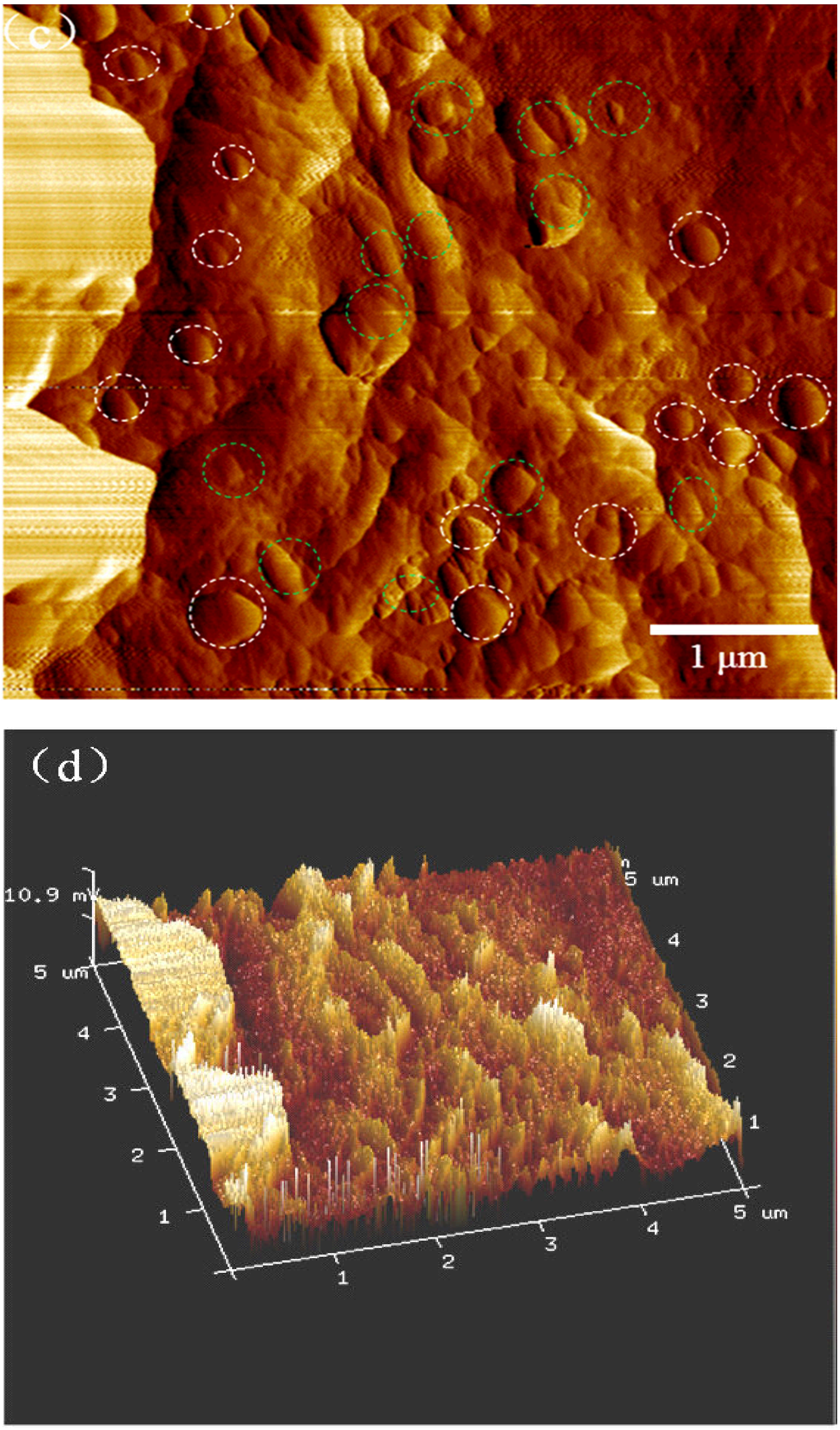
(c)
1 μm
(d)
10.9 mV
5 um
4
3
2
1
5 um
4
3
2
1
1
2
3
4
5 um

Figure S3 FT-IR spectra of the sugarcane bagasse (a) before and (b) after the AGO pretreatment performed at 220 °C for 2 h with a stirring speed of 150 rpm

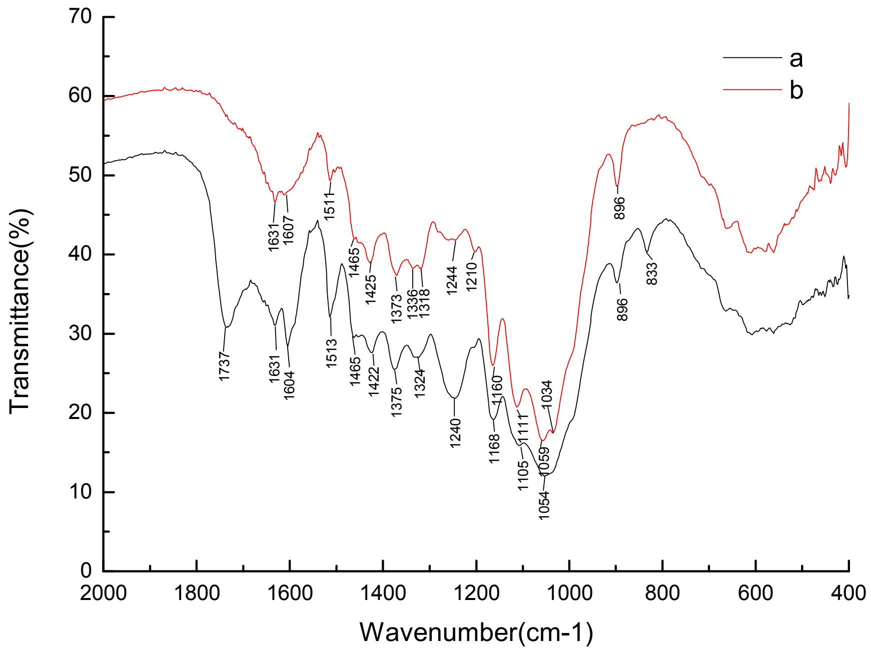

Supplement: Supplementary file 1 — 10.1186/s13068-016-0472-7 The substrate roughness measured from AFM images. Figure S1. SEM images of the sugarcane bagasse before and after the AGO pretreatment. Figure S2. AFM images of the sugarcane bagasse before and after the AGO pretreatment. Figure S3. FT-IR spectra of the sugarcane bagasse before and after the AGO pretreatment. [file 13068_2016_472_MOESM1_ESM.pdf]
